# Supplementary material for: Rapid turnover of life-cycle-related genes in the brown algae
Source: Genome Biol. 2019 Feb 14;20:35. doi: 10.1186/s13059-019-1630-6 (PMC6374913; doi:10.1186/s13059-019-1630-6)
Supplement: Supplementary file 3 — Protein alignments, corresponding Gblocks html file and CDS sequences for the lineage conserved gametophyte-biased genes. (ZIP 2109 kb) [file 13059_2019_1630_MOESM3_ESM.zip › Con_GA_alignments/Ec-17_000540.aa_ali.fasta-gb.htm]

Ec-17\_000540.aa.fasta.trans.aa\_ali.fasta


## Gblocks 0.91b Results

Processed file: **Ec-17\_000540.aa.fasta.trans.aa\_ali.fasta**  
Number of sequences: **2**  
Alignment assumed to be: **Protein**  
New number of positions: **378** (selected positions are underlined in blue)

```
                         10        20        30        40        50        60
                 =========+=========+=========+=========+=========+=========+
Ec-17_000540     MMAAAGRESHRSTSAERTLLSPPGRESEEGAERSRAADDESIFGGDPAGFGAGCEAGVGN
MSTRG.28242.1    ------------------------------------------------------------
                                                                             


                         70        80        90       100       110       120
                 =========+=========+=========+=========+=========+=========+
Ec-17_000540     VEALYSDQQTAPRAKKPAKRGRRKNKSSPGGPSEVAAAIDTTGGDVPAATSAAASEGEQG
MSTRG.28242.1    ------------------------------------------------------------
                                                                             


                        130       140       150       160       170       180
                 =========+=========+=========+=========+=========+=========+
Ec-17_000540     GEVLDAPPAGFPAPVAREDGGLDGDGMESARTTSADKPATRERSQQMYEKHRQTQEKLRA
MSTRG.28242.1    ------------------------------------------------------------
                                                                             


                        190       200       210       220       230       240
                 =========+=========+=========+=========+=========+=========+
Ec-17_000540     KRDETATSGCTFAPQLSSKQKGRKSEEAVTSDHLYKDFNKQLEKRERLAQEESKKLSFRP
MSTRG.28242.1    -------------------------------------------KRERLAEEERQKISFRP
                                                            #################


                        250       260       270       280       290       300
                 =========+=========+=========+=========+=========+=========+
Ec-17_000540     VLPTASSRLAAKMSATTP-VEGHKRAEDKSKATAEAMYKECTFSPDTVVTKALTKKILAA
MSTRG.28242.1    VLPTASSRLAAKIAATDPSVDGHKRAEDKSKATAEAMYKECTFAPNTVVTKPLTEKILAA
                 ################## #########################################


                        310       320       330       340       350       360
                 =========+=========+=========+=========+=========+=========+
Ec-17_000540     KGKQAPVADRLYATPETQAALAKSREEARAAKELAACTFRPDTSGSSKRSSAGSGRRDSA
MSTRG.28242.1    KGKQAEVADRLYASAETQAALAKTREEARVAKELAACTFRPDTSGSSKRSSAGSGRGDSG
                 ########################################################### 


                        370       380       390       400       410       420
                 =========+=========+=========+=========+=========+=========+
Ec-17_000540     GNTNTHDRLFGLHGEMLMTKQAKREELATKERMTCTFSPALVAKHVPKG------GKKLL
MSTRG.28242.1    ---NTHERLFGLHGELLMSKQTKKEELAARELMECTFAPALVAKSVVPSSSDSDKGVKNA
                    ###########################################              


                        430       440       450       460       470       480
                 =========+=========+=========+=========+=========+=========+
Ec-17_000540     CVDTEN------------EPVYERLYSARGRAQAREAFVDPECTFQP--HFKSKSVSSSP
MSTRG.28242.1    STDKDAAARGSATGAERAESIHERLYKLRVRAEASVEFLEPECTFQPDLALTNKSLASAR
                                   #############################             


                        490       500       510       520       530       540
                 =========+=========+=========+=========+=========+=========+
Ec-17_000540     R--RRDNPEESWADAMYKLGKERQTARSQSPRNYTATFRDQDEAKEMQLCTFQPDVSSTQ
MSTRG.28242.1    CRGKTEDKGERRMDVLYKHGRQRQAARSQSPRNYSAKFREDDESKEMELCTFQPNVSSTQ
                          ###################################################


                        550       560       570       580       590       600
                 =========+=========+=========+=========+=========+=========+
Ec-17_000540     NSRFKAGSRGGALFDTLYAKHVDKTNARERVREEDMKACTFSPQLASHKTRMGRSLSPQR
MSTRG.28242.1    TGRFKTESRGKALFATLYGQRGDRDRARDRAWKEDMKACPFSPTLSSHKTRVGRSLSPRR
                 ############################################################


                        610       620       630       640       650       660
                 =========+=========+=========+=========+=========+=========+
Ec-17_000540     QGPVYEELYKEASAQRFSRTLSAEHLQSQQCPFKPQLATRRRSPQATSSTKAPPAATAGG
MSTRG.28242.1    ERPVYEELYGEAATQRLARTMSAEYIQSQECPFKPNMATARSRTSRAGTSSATRASIEER
                 #########################################          #########


                        670       680       690       700       710       720
                 =========+=========+=========+=========+=========+=========+
Ec-17_000540     GQGEAGAQSPLDTSSDDAAAAVAEGTSDVDVALPSVPGSLPSVQQSMGPPAQGSETHATG
MSTRG.28242.1    RQASPLSQPP--------------------------------------------------
                 ##########                                                  


                        730       740       750
                 =========+=========+=========+======
Ec-17_000540     GDGATASPPLPPPVGDEEEDEQEGGGEGEGQEVYDY
MSTRG.28242.1    -------------SSQDHVAASTPAAAVIAAAATAX
```

```
Parameters used
Minimum Number Of Sequences For A Conserved Position: 2
Minimum Number Of Sequences For A Flanking Position: 2
Maximum Number Of Contiguous Nonconserved Positions: 8
Minimum Length Of A Block: 10
Allowed Gap Positions: None
Use Similarity Matrices: Yes
```

```
Flank positions of the 6 selected block(s)
Flanks: [224  258]  [260  359]  [364  406]  [439  467]  [490  641]  [652  670]  

New number of positions in Ec-17_000540.aa.fasta.trans.aa_ali.fasta-gb:  378  (50% of the original 756 positions)
```
